# Learning performance and brain structure of artificially-reared honey bees fed with different quantities of food

**DOI:** 10.7717/peerj.3858

**Published:** 2017-10-23

**Authors:** Karin Steijven, Johannes Spaethe, Ingolf Steffan-Dewenter, Stephan Härtel

**Affiliations:** 1 Department of Animal Ecology & Tropical Biology, University of Würzburg, Würzburg, Germany; 2 Lectorat Bee Health-Domain Animals and Business, Van Hall Larenstein, University of Applied Sciences, Leeuwarden, Netherlands; 3 Department of Behavioral Physiology & Sociobiology, University of Würzburg, Würzburg, Germany

**Keywords:** Nutrition, Mushroom bodies, Proboscis extension reflex, Confocal laser scanning microscopy, Morphometry, *Apis mellifera*, Brain development, Differential olfactory conditioning, Neuroanatomy, Cognition

## Abstract

**Background:**

Artificial rearing of honey bee larvae is an established method which enables to fully standardize the rearing environment and to manipulate the supplied diet to the brood. However, there are no studies which compare learning performance or neuroanatomic differences of artificially-reared (in-lab) bees in comparison with their in-hive reared counterparts.

**Methods:**

Here we tested how different quantities of food during larval development affect body size, brain morphology and learning ability of adult honey bees. We used in-lab rearing to be able to manipulate the total quantity of food consumed during larval development. After hatching, a subset of the bees was taken for which we made 3D reconstructions of the brains using confocal laser-scanning microscopy. Learning ability and memory formation of the remaining bees was tested in a differential olfactory conditioning experiment. Finally, we evaluated how bees reared with different quantities of artificial diet compared to in-hive reared bees.

**Results:**

Thorax and head size of in-lab reared honey bees, when fed the standard diet of 160 µl or less, were slightly smaller than hive bees. The brain structure analyses showed that artificially reared bees had smaller mushroom body (MB) lateral calyces than their in-hive counterparts, independently of the quantity of food they received. However, they showed the same total brain size and the same associative learning ability as in-hive reared bees. In terms of mid-term memory, but not early long-term memory, they performed even better than the in-hive control.

**Discussion:**

We have demonstrated that bees that are reared artificially (according to the Aupinel protocol) and kept in lab-conditions perform the same or even better than their in-hive sisters in an olfactory conditioning experiment even though their lateral calyces were consistently smaller at emergence. The applied combination of experimental manipulation during the larval phase plus subsequent behavioral and neuro-anatomic analyses is a powerful tool for basic and applied honey bee research.

## Introduction

Laboratory rearing of honey bee workers has become a very powerful tool in various fields within honey bee research. It is used to study questions related to caste development and physiology (e.g., De Souza et al., 2015; Kucharski et al., 2008; Von Rhein, 1933; Weaver, 1955), and since honey bee colonies started to show distressing declines, this method is increasingly used to tackle questions related to larval pathogens (e.g., Foley et al., 2012; Genersch, Ashiralieva & Fries, 2005; Peng et al., 1996), ecotoxicology (e.g., Charpentier et al., 2014; Hendriksma, Härtel & Steffan-Dewenter, 2011a; Medrzycki et al., 2010), and biosafety of transgenic plants (e.g., Brodsgaard et al., 2003; Hendriksma, Härtel & Steffan-Dewenter, 2011a; Hendriksma, Härtel & Steffan-Dewenter, 2011b; Li et al., 2014; Steijven, Steffan-Dewenter & Härtel, 2015 also see Crailsheim et al., 2013 for a more exhaustive review). In recent years, most labs use the method described by Aupinel et al. (2005) and Aupinel et al. (2007). In a ring test that was carried out at seven laboratories, a high level of standardization of this method was attained (Aupinel et al., 2009) and since then different groups have sought to further improve the protocol (Crailsheim et al., 2013; Hendriksma, Härtel & Steffan-Dewenter, 2011a; Lüken et al., 2014). However, despite the effort to standardize rearing protocols, surprisingly few studies exist that evaluate the ‘quality’ of bees obtained via this artificial rearing method. To our knowledge there is only one peer reviewed publication that showed that artificially-reared bees are comparable with their in-hive reared sisters in terms of flight performance (Brodschneider, Riessberger-Gallé & Crailsheim, 2009). Other works from the same group have shown that artificially-reared bees do not differ in weight or protein content from their in-hive counterparts (Riessberger-Gallé, Vollmann & Crailsheim, 2008; Riessberger-Gallé et al., 2008). However, whether artificial diet and rearing under lab conditions affect the brain or cognitive capabilities is completely unknown.

The larval and pupal phase is the most vulnerable stage of a honeybee’s development. For example, only small deviations from the optimal rearing temperature during pupal development cause serious defects in the neuronal brain architecture and learning performance of honeybee workers (Groh, Tautz & Rossler, 2004; Tautz et al., 2003). In previous projects (K Steijven, 2015, unpublished data) we noticed that bees reared in the lab were somewhat smaller than the bees found in hives (also see Brodschneider, Riessberger-Gallé & Crailsheim, 2009). We thus aimed at evaluating whether a reduction in size could also have an effect on brain development and the cognitive abilities of bees. A particular brain structure that is known to be important for learning and memory is the mushroom body (MB; De Belle & Heisenberg, 1994; Dubnau et al., 2001; Heisenberg, 1998; Heisenberg et al., 1985; Kaulen, Erber & Mobbs, 1984; Menzel, 1999). In honey bees, as well as other social insects, the MB is enlarged in the forager caste compared to their in hive nest mates (e.g., Fahrbach et al., 1998; Withers, Fahrbach & Robinson, 1993), which is probably coupled with the need to perform complex behaviors that require increased cognitive abilities such as, for example, locating food sources, navigating in complex landscapes, and communicating the location of preferred resources to nest mates.

Manipulation of the larval diet under in-hive conditions is confounded by a number of colony related factors. In particular the administration of different food quantities to the larvae under in-hive conditions is hard to achieve since nurse bees would interfere to supply the same quantity of food to each of the tested larvae (Mattila & Smith, 2008). To study the effects of different quantities of food on brain development and the cognitive abilities of bees, one has to rear workers artificially. We experimentally varied the quantity of food given to artificially-reared bees and compared their body size and the size of their brain neuropils (including MBs) with in-hive reared bees. Additionally we examined the cognitive traits, learning performance and memory formation, in a differential olfactory conditioning experiment (for an overview see Giurfa & Sandoz, 2012). With this, we aimed to evaluate whether artificially-reared bees are qualitatively comparable to in-hive reared bees in terms of their cognitive abilities.

## Material & Methods

### Artificial rearing

We reared 853 worker bees artificially under four different feeding regimes (Table 1) that varied in total quantity of artificial diet fed; 150, 160, 170, and 180 µl of total food volume. Rearing took place from the 10th of April 2014 until the 2nd of May 2014 (date of eclosion). Worker bees were obtained from two hives in our institutional apiary at the Biocenter, University of Würzburg. We controlled for age of the bees by enclosing queens on an empty brood comb for 24 h (April 10, 2014) using a wire mesh cage that allowed nurse bees to freely pass, but kept queens confined. Upon hatching of the eggs (April 14, 2014), combs were transferred to the lab, where individual larvae were grafted and placed in a small plastic cup (Nicotplast, Maisod, France) containing 20 µl of artificial diet, situated in a sterile 48-well plate. Well plates and content were pre-heated to 35 °C and grafting took place on a thermal mat (ThermoLux, Murrhardt, Germany), to avoid hypothermia. Well plates were kept in a brood stove at 35 °C and 95% RH. Larvae were reared according to the protocol of (Aupinel et al. (2005); Aupinel et al. (2007) and Aupinel et al. (2009) also see Crailsheim et al., 2013) with some adjustments in regard to the total quantity of artificial diet being fed. We fed four different quantities of artificial diet; 150 µl, 160 µl (according to Aupinel), 170 µl and 180 µl. We varied the quantity of diet given on the last two days to obtain the described gradient (Table 1). Each treatment was represented on each well plate. After pupation well plates were placed in another brood stove with 35 °C and 80% RH. Prior to eclosion plastic cups containing pupae were reordered so that well plates contained only pupae with the same hive origin, that received the same treatment, and these were placed in sealed hatching boxes. From each hive we collected two capped brood combs and also placed them in the incubator one day before artificially-reared bees were starting to eclose (May 2, 2014), we marked hatching adults from these combs and placed them back in their hive for the in-hive control. Hatched in-lab bees were collected and placed in flight cages, kept in a brood stove (28 °C, 70% RH) until the olfactory conditioning experiment 11 days after hatching. Upon eclosion we took a subsample of 10 bees from each treatment (the in-lab reared treatments, as well as the in-hive control group) for brain reconstruction.

**Table 1:** Feeding regime for the different treatment groups. All larvae were reared according to the protocol described by Aupinel* (2005, 2007, 2009) with slight variations in the last two feeding days (Day 8, Day 9) that resulted in differing total quantities of food.

Day 4	Day 5	Day 6	Day 7	*Day 8*	*Day 9*	
Diet A	(Diet A)	Diet B	Diet C	*Diet C*	*Diet C*	**Total**
20 µl	–	20 µl	30 µl	40 µl	*40 µl*	150 µl
20 µl	–	20 µl	30 µl	40 µl	50 µl	160 µl*
20 µl	–	20 µl	30 µl	*50 µl*	50 µl	170 µl
20 µl	–	20 µl	30 µl	*50 µl*	*60 µl*	180 µl

### Olfactory conditioning

We performed a classical differential olfactory conditioning experiment (see Matsumoto et al., 2012). Conditioning took place from the 12th until the 16th of May and again from the 20th until the 22nd of May 2014. Mean age of the conditioned bees was 16 (±5) days. On the day prior to conditioning bees were immobilized on ice and placed in a plastic harness tube. We let the bees get accustomed to the new situation for about half an hour and fed them until satiation with a 50% (w/w) sucrose solution. Bees were kept in a brood stove (23 °C, 70% RH) and starved overnight until the conditioning trials the following day, in order to establish a uniform hunger level. Half an hour prior to the first conditioning trial bees were taken out of the brood stove and tested for a sucrose response by touching their antennae with a 50% sucrose solution. The proportion of bees that failed to produce PER at the start of the conditioning trials was used as a proxy for sucrose responsiveness which is known to correlate with learning performance in honey bees (Scheiner, 2004; Scheiner, 2012; Scheiner et al., 2005). Bees that showed proboscis extension reflex (PER) were placed in compartments on a rail that slides in front of a ventilation system. We used two different odors as conditioned stimulus (CS); 1-nonanol as the rewarded stimulus (CS+), and 1-hexanol as the unrewarded stimulus (CS−). The reward, or unconditioned stimulus (US), was a 50% (w/w) sucrose solution. Harnessed bees were subjected to twelve conditioning trials in which CS+ and CS− were presented in a haphazard order (each odor six times), followed by two retention tests; one hour, and 48 h after the last trial. Retention tests were carried out on the same bees. We conducted conditioning (and retention tests) as a blind study. To present the CS we used two 20 ml syringes, each with a piece of filter paper that contained 4 µl of either odor. The syringe was held about 15 mm from the bees’ antennae and CS was presented for four seconds. Two seconds after the onset of CS+ the US was presented for 1 s with a toothpick to the antennae to elicit PER, after which the toothpick was held to the proboscis for two seconds. When using CS- a toothpick was held in front of the bee, so that the visual cues were the same as with CS+, but bees were not rewarded. We waited some seconds to allow residual odors to be removed by the ventilation system behind the bee, then moved the next bee in front of the ventilator. After a 10 s pause conditioning was performed on the next bee. Between trials was a 10 min interval. If the bee showed PER to the CS+ the response was recorded as 1, no response was recorded as 0. If the bee showed no PER upon antennal contact it was recorded as n, so that bees that did not show PER three times upon antennal contact could be excluded from the experiment. For the statistical analysis n was considered as a 0. Bees that showed spontaneous PER in the first CS+ trial within the first 2 s were also excluded from the experiment. One hour after the 12th trial, response to CS+, CS−, and US were tested (1 h retention test). CS+ and CS− were tested in a similar fashion as in the conditioning phase but without the reward, US was presented to assure that bees were still capable of PER. If not, those bees were also excluded from the experiment. Bees were kept confined in their tubes and fed to satiation regularly. This retention test was then repeated one more time after 48 h after last conditioning trial. After the last retention test bees were frozen and stored at −20 °C for further measurements. To compare bee size over feed treatments we measured head size and intertegulae span (ITS) using an imaging software program (ImageJ; US National Institutes of Health, Bethesda, MD, USA). To determine head size the shortest distance between the compound eyes was measured, and for ITS the shortest distance between the tegulae was taken (Cane, 1987). As an additional control, we conducted a reciprocal conditioning experiment to show that the bees can associate either odor with the presence or absence of a reward. Conditioning took place from the 22nd until 28th of October 2015. Another 168 bees were taken from the same hives as we used for the initial olfactory conditioning experiment. Half of the bees (82 bees) were conditioned using 1-nonanol as CS+ and 1-hexanol as CS−, for the other half (86 bees) 1-hexanol was used as CS+ and 1-nonanol as CS−.

### Wholemount preparations

To investigate possible effects of diet on neuronal development, we compared volume of brain neuropils of freshly eclosed bees from all five treatment groups. Brains were prepared according to the protocol by Muenz et al. (2015). In brief, the bee was anaesthetized on ice, the head was removed and transferred to a wax dish. The head was then covered with physiological saline (130 mM NaCl, 5 mM KCl, 4 mM MgCl_2_, 5 mM CaCl_2_, 15 mM Hepes, 25 mM glucose, 160 mM sucrose; pH 7.2) and a small window was cut into the frons between the antennae. After removing glands and tracheae the brain tissue was immediately transferred into ice-cold 4% formaldehyde (FA; methanol free, 28908; Fischer Scientific, Schwerte, Germany) in 0.01 M phosphate-buffered saline (PBS; pH 7.2) and fixed at 4 °C overnight. For visualization of different neuropils, we applied an anti-synapsin I antibody in whole mount preparations following the protocol by Groh et al. (2012). The fixed brain was treated with PBS and PBS Triton X-100, before being incubated with the primary mouse antibody SYNORF1 (1:10; kindly provided by E. Buchner, University Würzburg, Germany). A CF488A conjugated goat anti-mouse antibody (1:250; Biotrend, Köln, Germany) was applied for fluorescence labeling and the brain was then dehydrated in an ascending ethanol series and mounted on custom made metal slides in methyl salicylate (M-2047; Sigma Aldrich, Steinheim, Germany).

### Confocal laser-scanning microscopy and 3D-reconstructions

**Figure 1:**
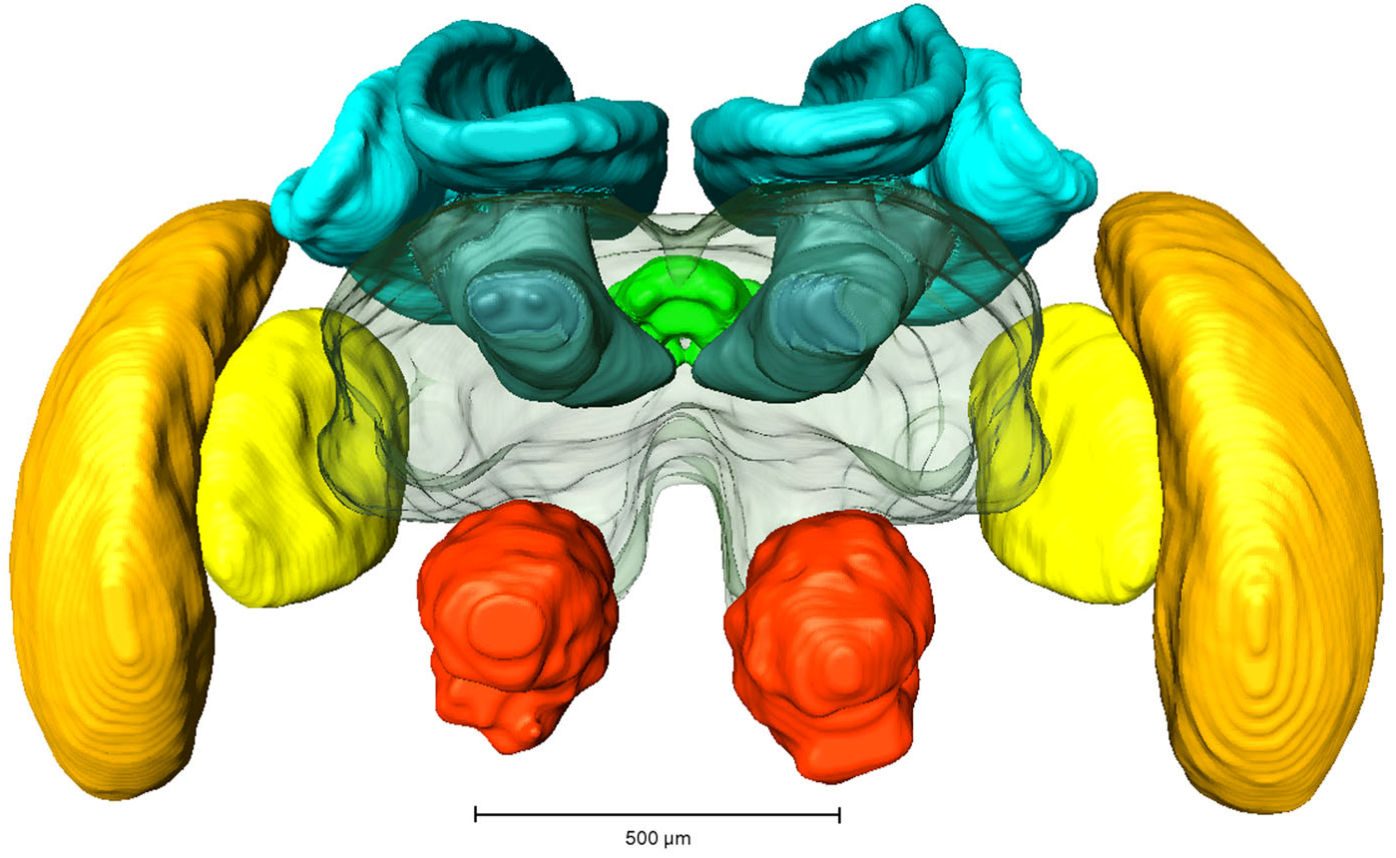
Reconstruction of a brain from an in-lab reared worker. Red, Antennal lobes, AL; Blue, Mushroom bodies, MB (comprising medial and lateral calyx and the pedunculus); Green, central complex, CB (comprising upper and lower central complex and protocerebral bridge); Yellow, Lobula; Orange, Medulla.

Whole mount preparations of synapsin labeled brains were scanned using a laser-scanning confocal microscope (Leica TCS SP2 AOBS; Leica Microsystems AG, Wetzlar, Germany) equipped with an argon/krypton and three diode lasers. A HC_PL_APO objective lense (10x/0.4 NA imm) was used for image acquisition and optical sections were taken in 5 µm steps. Image processing was performed using 3D image software AMIRA 5.3 (FEI Visualization Group, Mérignac, France, see Fig. 1). Based on outline and interpolation reconstructions the volumes of the following neuropils (without cell bodies) were measured (also see Fig. 1): antennal lobe (AL), optic lobe (OL; comprising the medulla and lobula), mushroom body (MB, comprising calyx and pedunculus), central complex (CX, comprising upper and lower central body and protocerebral bridge) and protocerebrum (PC, without suboesophageal ganglion). Volumetric data were calculated in Amira 5.6 and exported to Microsoft Excel 2007 (Microsoft Corporation, Redmond, WA, USA) for further analysis.

### Statistical analysis

All statistics were performed using R statistical software (R Core Team, 2014). We evaluated whether there is a difference in the proportion of bees among treatment groups that failed to show PER at the start of the conditioning trials using a Chi-squared test. The responses during the conditioning trials (learning phase) were analyzed with a generalized linear mixed effects model (glmer function, binomial family) using the lme4 package (Bates et al., 2014). Preliminary analyses showed that on one of the conditioning days (14th of May 2014) bees were not able to learn well, probably due to a defective sink in the lab that caused a pungent smell. We decided to exclude these bees from further analysis. In the full model we specified PER as a binary variable in response to the fixed factors trial, CS (CS+ or CS−), and treatment (150 µl, 160 µl, 170 µl, 180 µl, and the control; in-hive), with their interactions (also see Kirkerud et al., 2013). To account for the differences in age of the conditioned bees as the experiment progressed, we included date (of conditioning) as random factor. Colony background was also included as random factor to account for potential differences between the two colonies that were used. Finally also bee ID was included as random factor to correct for the repeated measurements. Model simplification was carried out via stepwise model simplification using Likelihood Ratio Tests (LRT). Trial was no longer taken into account. The 1 h and 48 h retention tests (memory formation) were analyzed in a similar way; whether or not bees showed PER as the binary response variable, in relation to the factors CS and treatment, and their interaction, with colony background and date of conditioning in the random structure. Not all bees survived the 48 h period being harnessed in the plastic tubes, in two of the treatment groups (160 µl and 180 µl) none of the remaining bees responded to the CS−, resulting in unbalanced data. Thus for the 48 h retention test we used the function bglmer from the package blme (Dorie, 2014) which is able to work with unbalanced data. All models were checked for overdispersion. For the 1 h retention test we had overdispersion, which was dealt with by including an observation level random factor (Pirk et al., 2013).

To compare ITS and head width among treatment groups we carried out linear mixed effects models (function lme), including colony as random factor (Pinheiro et al., 2014). Visual evaluation of the model fits showed that residuals were normally distributed and the assumption of heteroscedasticity was met. For post hoc comparisons among treatment levels we carried out a Tukey test with Benjamini Holmes correction (Benjamini & Yekutieli, 2001).

Total brain volume, as well as the different neuropils (see previous paragraph) were analyzed with a regression type analysis (function lm, package stats—R Core Team, 2014) with treatment as only fixed factor. As all bees in the subsample were taken on the same day (right after eclosion) and originated from the same colony there was no need to specify a random structure here. Model validation was carried out via visual evaluation of the model residuals. All models had met the assumption of normal distribution of residuals, however the variances of all but one model (CB) were heterogeneous. For those we allowed for unequal variances across treatment groups using the function varIdent from the package nlme (Pinheiro et al., 2014; Zuur et al., 2009).

## Results

### Differential olfactory conditioning

**Figure 2:**
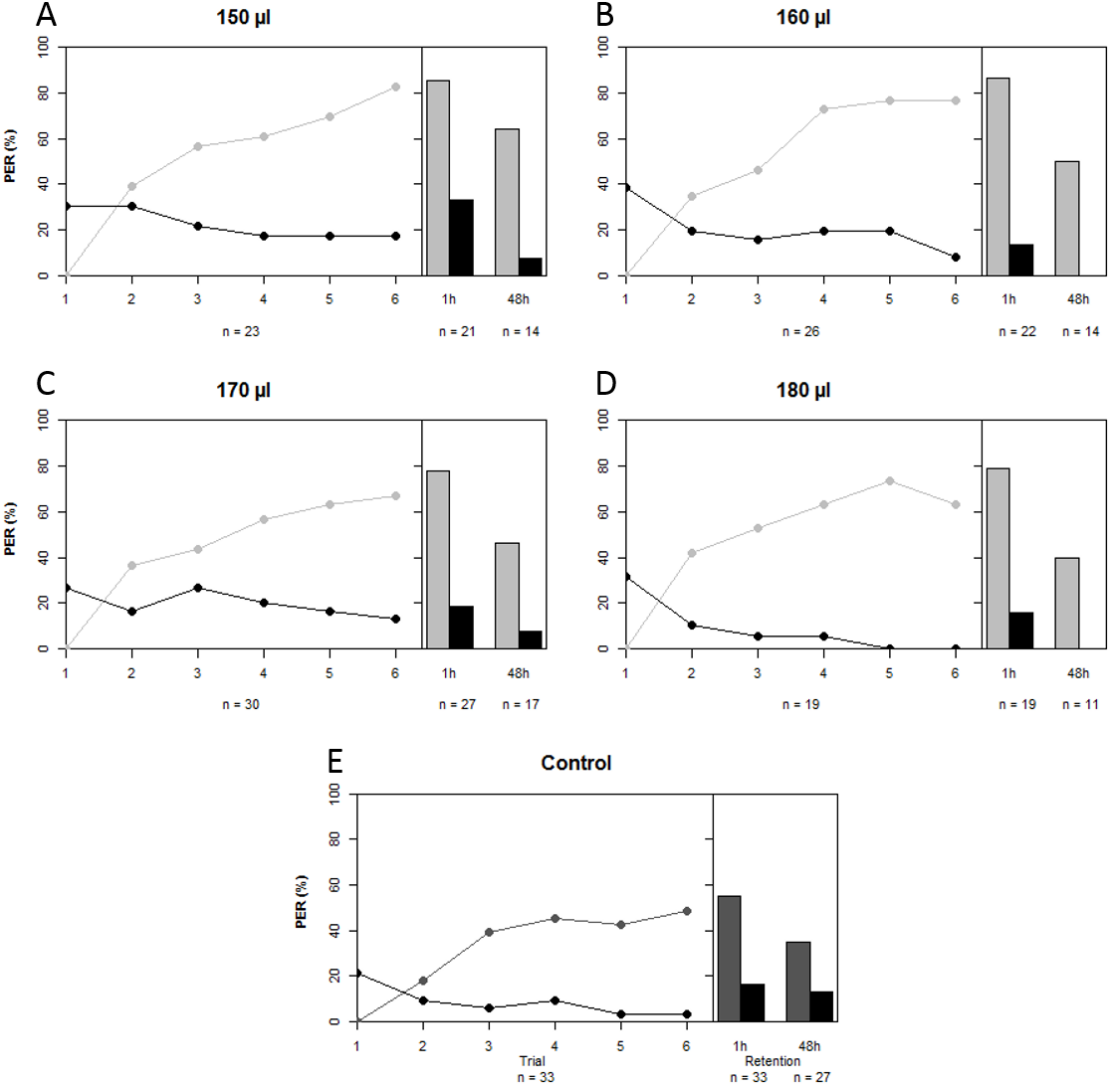
Learning curves and retention tests of the five treatment groups. Grey depicts the CS+ and black the CS−, light grey corresponds to the treatment groups that were reared in the lab with different quantities of total brood food consumed (A–D), and dark grey is the in-hive control (E). See text for statistics.

A total of 229 bees lived after being harnessed and starved for a night prior to the conditioning experiment. We compared the proportions of bees that failed to show PER at the start of the experiment among treatment groups as a proxy for sucrose responsiveness. There was no difference in sucrose responsiveness among treatment groups (χ^2^ = 7.472, *df* = 4, *p* = 0.113). A total of 140 bees were conditioned, seven of which had to be excluded because their behavior was influenced by a disturbance of the experiment (on 14th of May 2014). A further five bees that showed spontaneous PER upon the first trial were also removed from the data set, leaving a total of 128 conditioned bees to do the analysis with. In all artificially-reared groups, regardless of treatment, a majority of bees was able to differentiate between CS+ and CS− over the course of the conditioning period (GLMM: *z* = 12.200, *p* < 0.001). Upon first glance it seems that artificially-reared bees performed slightly better than bees that were reared in the hive (Fig. 2); the percentages of bees that showed PER to CS+ at the last trial were 83% (150 µl), 77% (160 µl), 67% (170 µl), 63% (180 µl), compared to 48% of the in-hive bees. However, statistically all groups that were reared artificially (regardless of the quantity of artificial food they received) performed equally well as the in-hive bees (LRT: χ^2^ = 6.600, *p* = 0.159). When we disregard trial, we find that those bees that received the highest quantity of food in the lab performed marginally better than the in-hive bees (GLMM: *z* = 1.796, *p* = 0.073), which can also be easily seen in the graph as none of those bees respond to the CS− as from trial 5. After one hour (mid-term memory; MTM) all bees that were reared in the lab perform better at differentiating CS+ and CS− than the in-hive control (GLMM: (150 µl) *z* = 1.982, *p* = 0.048; (160 µl) *z* = 2.076, *p* = 0.038; (170 µl) *z* = 4.662, *p* = 0.023, (180 µl) *z* = 1.999, *p* = 0.046). During the 48 h retention test (early long-term memory, eLTM) all treatment groups performed the same again (LRT: χ^2^ = 2.950, *p* = 0.566). During the reciprocal conditioning bees showed to be able to associate either odor equally well with the presence or absence of a reward (GLMM (CS+ = 1-nonanol): *z* = 9.682, *p* < 0.001; (CS+ = 1-hexanol): *z* = 9.819, *p* < 0.001), as well as distinguish between odors regardless which odor was used as CS + during conditioning after 1 h (GLMM: (CS+ = 1-nonanol): *z* = 9.184, *p* < 0.001; (CS+ = 1-hexanol): *z* = 7.766, *p* < 0.001) and after 48 h (GLMM: (CS+ = 1-nonanol): *z* = 18619, *p* < 0.001; (CS+ = 1-hexanol): *z* = 3.486, *p* < 0.001). For a graphical depiction of these outcomes see the Supplemental Information.

### Morphometrics

**Figure 3:**
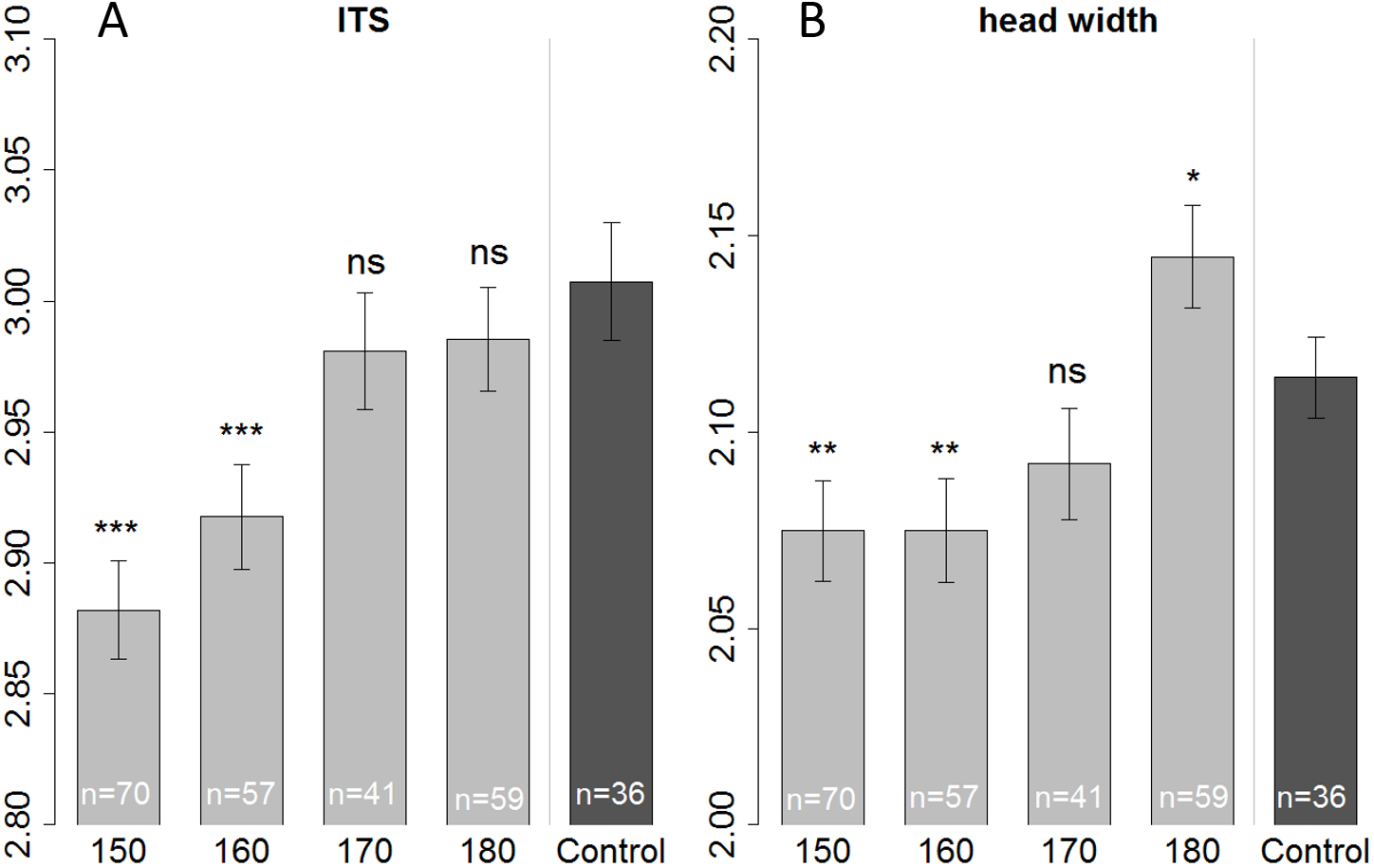
Morphometric measurements of ITS (inter tegula span; A) and head width (B). All treatment groups that were reared in the lab (with 150, 160, 170, and 180 µl of artificial diet) were compared to the in-hive control (*x*-axis), size is depicted in mm (*y*-axis). Asterisks depict significance levels (*p* < 0.05, ∗; *p* < 0.01, ∗∗; *p* < 0.001, ∗∗∗; ns, not significant).

We measured head and thorax width (ITS) of all the bees that were used for the olfactory conditioning experiment, plus some additional bees (that were not suitable for conditioning, or had died before start of the experiment) resulting in a total number of 263 bees. When fed with 150 or 160 µl bees were smaller than those of the control in terms of ITS (Linear regression: (150 µl) *t* =  − 6.687, *p* < 0.001; (160 µl) *t* =  − 4.493, *p* < 0.001) as well as head width (Linear regression: (150 µl) *t* =  − 3.073, *p* = 0.002; (160 µl) *t* =  − 2.954, *p* = 0.003). But bees fed with only 150 µl tend to be even smaller (ITS) than bees fed with 160 µl (Fig. 3—not significant; Tukey post hoc test: *z* = 2.219, *p* = 0.170), yet their heads had the same size (Tukey post hoc test: *z* = 0.008, *p* = 1.000). There seems to be a tradeoff; with limiting resources a bee invests in the development of the head, at cost of body size (ITS). Likewise, when fed with a surplus diet (180 µl treatment) bees’ heads were significantly bigger than the in-hive control (linear regression: *t* = 2.343, *p* = 0.020) yet in terms of ITS, bees had the same size as the control (linear regression: *t* =  − 1.107, *p* = 0.269).

**Figure 4:**
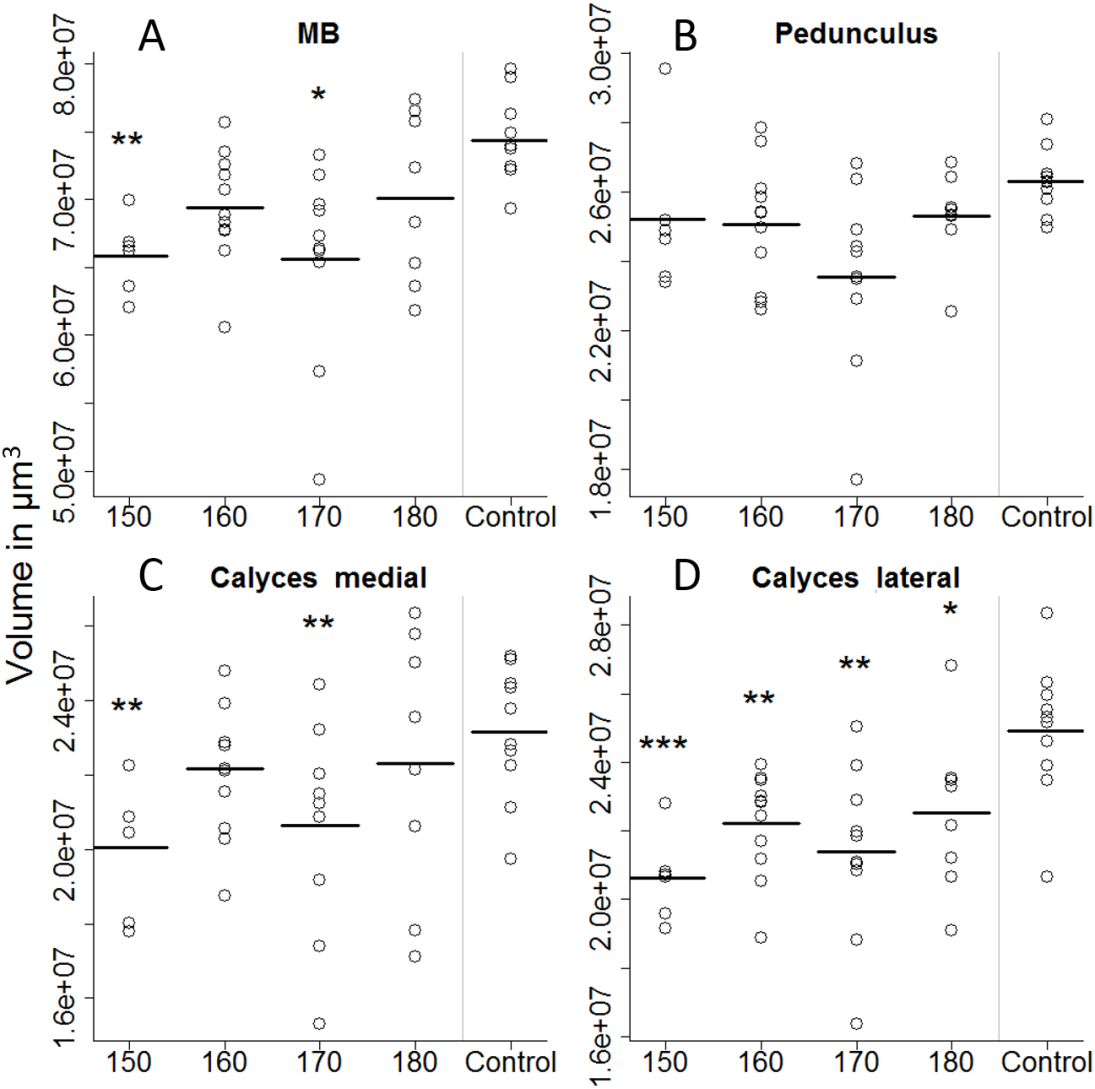
Volumetric reconstruction of the mushroom bodies (MB, A), containing the pedunculus (B) and the medial (C) and the lateral calyces (D). All treatment groups that were reared in the lab (with 150, 160, 170, and 180 µl of artificial diet) were compared to the in-hive control. Asterisks depict significance levels (*p* < 0.05, ∗; *p* < 0.01, ∗∗; *p* < 0.001, ∗∗∗).

On the first day of eclosion, 10 bees per treatment group were taken as a subsample for brain volume reconstruction. Out of those 50, 45 bee brains were successfully reconstructed (Fig. 4). Looking at the overall brain volume, we did not detect any differences (Table 2). However the brain sizes of bees that were fed between 150 and 170 µl were marginally smaller than the control. Looking in more detail, we find that artificially-reared bees had smaller calyces. For the 150 and 170 µl groups medial calyces were smaller than the in-hive control and lateral calyces were consistently smaller for all treatment groups (Table 3). This indicates that higher central neuropils (MB) but not the periphery (OL and AL) have been affected.

## Discussion

We investigated how well artificially-reared bees perform compared to in-hive reared bees in terms of learning and memory formation. We fed different quantities of artificial diet and conducted a differential olfactory conditioning experiment. Additionally we measured morphometric parameters, head width and ITS, and compared the brain morphology among treatment groups. As treatment groups did not differ in terms of sucrose responsiveness, it can be assumed that any differences found in the behavioural experiment relate to differences in learning/memory formation rather than differences in appetitive motivation. Even though bees reared in the lab with the standard quantity of artificial food, i.e., 160 µl as described by Aupinel et al. (2005); Aupinel et al. (2007) and Aupinel et al. (2009), were smaller and had smaller lateral MB calyces than their in hive sisters, they did perform equally well in the olfactory conditioning experiment. Note that we reconstructed brains directly after hatching (Day 1) and that we conducted the conditioning experiment at a later time (Day 11–Day 21), hence there is no causal relation between the size of the MBs and the learning performance of the bees. Honey bee brains are known to be plastic, and their calyces do become larger as they get older and more experienced (Durst, Eichmuller & Menzel, 1994; Maleszka et al., 2009; Withers, Fahrbach & Robinson, 1993; Withers, Fahrbach & Robinson, 1995; Fahrbach & Nest, 2016). We set out to evaluate the effect of in-lab rearing as well as the potential effect of quantity of larval food, thus we reconstructed brains immediately after hatching. Since all treatment groups reared in the lab had consistently smaller lateral calyces (and smaller medial calyces for the 150 µl and 170 µl group) than the in-hive group, we can conclude that the process of in-lab rearing does have an effect on brain development. It might very well be that artificially-reared bees ‘catch up’ with their in hive sisters in the 11 to 21 days until the conditioning experiment took place, as all in-lab treatment groups learned to differentiate between the two scents (CS+ and CS−) equally well (Fahrbach, 2006; Farris, Robinson & Fahrbach, 2001). We know from a recent study that bees with anesthetized MBs are able to perform elemental olfactory discriminations, but not configural ones (Devaud et al., 2015). Thus, the smaller MBs of the artificially-reared bees compared to those of their hive-reared counterparts might not affect the learning outcome of the elemental olfactory conditioning experiment we conducted. MTM (1 h retention) was slightly better for in-lab reared bees than that of the in-hive control group, but in terms of eLTM, (48 h retention) the treatment groups did not differ significantly. Note that the 48 h retention test was conducted on the same group of bees that was used to measure the 1 h retention. Possibly this could result in an extinction effect; however, previous studies have shown this extinction effect to be rather weak (Menzel et al., 2001; Wustenberg, Gerber & Menzel, 1998). The reciprocal conditioning experiment showed that both odors used (1-hexanol and 1-nonanol) could be associated with a reward equally well, which is in congruence with earlier studies (e.g., Carcaud et al., 2009; Vergoz et al., 2007).

**Table 2:** Linear model outputs looking at the effect of feeding treatment on brain size; different neuropils of the honey bee brains were reconstructed separately. Only in the mushroom bodies an effect of feeding treatment was detected. See Table 3 for the post hoc analysis. Asterisks depict significance levels (*p* < 0.05, ∗; *p* < 0.01, ∗∗; *p* < 0.001, ∗∗∗; NS, not significant).

Response variable	*F* value	*p* value	Sign.
Total volume	2.256	0.0806	.
Antennal lobes	0.621	0.6505	NS
Optical lobes	0.938	0.4517	NS
Medulla	0.712	0.5883	NS
Lobula	1.914	0.1269	NS
Mushroom body	3.273	0.0206	*
Pedunculus	0.876	0.4866	NS
Medial calyces	3.51	0.0151	*
Lateral calyces	7.174	0.0002	**
Central complex	1.1641	0.3411	NS

**Table 3:** Post hoc analyses of the effect of feeding treatment on mushroom body size. The different treatment levels of in-lab reared bees are compared with the control; the in-hive reared bees. First the results from the post hoc analysis for the entire mushroom body are depicted, followed by the results for neuropils that comprise the mushroom bodies; the penduculi, medial calyes and lateral calyces. The size estimates are depicted in µm^3^. The medial calyces of bees that received 150 µl or 170 µl of food, and the lateral calyces of all in-lab reared bees were smaller than the in-hive control. When significant differences to the control group were found results are depicted in bold.

	Treatment	Estimate	Std. Error	*t* value	*p* value	Sign.
Mushroom bodies	**150**	**65,861,460**	**2,336,384**	**−3.0819**	**0.0037**	**
	160	69,430,580	2,393,751	−1.5171	0.1371	NS
	**170**	**65,570,509**	**3,052,540**	−**2.4542**	**0.0186**	*
	180	70,123278	29,93615	−0.9817	0.3322	NS
Pedunculum	150	25,196,426	1,641,073	0.1246	0.9014	NS
	160	25,053,080	1,462,317	0.0419	0.9668	NS
	170	23,551,381	1,594,782	−0.9033	0.3718	NS
	180	25,294,705	1,434,007	0.2112	0.8338	NS
Medial calyces	**150**	**20,042,313**	**915,030**	−**3.3914**	**0.0016**	*
	160	22,157,112	78,0361	−1.2666	0.2126	NS
	**170**	**20,637,636**	**1,021,829**	**−2.4543**	**0.0186**	*
	180	22,298,411	1,370,218	−0.6183	0.5399	NS
Lateral calyces	**150**	**20,622,721**	**822,834**	−**5.2281**	**0.0000**	***
	**160**	**22,220,388**	**786,379**	−**3.4388**	**0.0014**	**
	**170**	**21,381,491**	**1,008,426**	−**3.5135**	**0.0011**	**
	**180**	**21,985,845**	**1,043,305**	−**2.2951**	**0.0271**	*

When faced with limiting food resources, it seems that bees invest in their heads at cost of body size. However, this trade-off is not reflected in their brain development. Similar results have been found by Brodschneider and coworkers, who showed that artificially-reared bees had smaller thoraces but same sized heads (Brodschneider, Riessberger-Gallé & Crailsheim, 2009). In in-hive reared honey bee workers, in contrast to bumble bee workers, little size variation exists. And where larger bumble bee foragers also have bigger brains, this correlation does not exist for honey bees (Mares, Ash & Gronenberg, 2005). We have shown experimentally that artificially-reared worker bees that were fed a range of total quantity of artificial diet, behave similar to hive bees in a differential olfactory conditioning experiment, even though they tend to be smaller when fed less. When fed more, especially their heads get bigger than the control group’s heads, but this is not true for their brains. And even though MBs are smaller upon hatching, we have shown that artificially-reared bees are comparable to in hive reared bees concerning relative simple cognitive tasks, like the differential olfactory conditioning experiment we conducted.

This opens up opportunities to reduce the natural variation in learning performance caused by parameters such as age, nutrition (in the larval and adult phase), rearing temperature (larval and pupal phase), and exposure to agrochemicals. In olfactory conditioning experiments typically foragers returning to the hive, or individuals caught from an artificial feeder are intercepted and used for conditioning, but these bees likely vary in age. How suitable a bee is for olfactory conditioning is highly age dependent (Laloi et al., 2001; Ray & Ferneyhough, 1997). In younger bees a higher percentage shows spontaneous PER (i.e., without conditioning), conditioning worked better for older bees (in one out of two tested odors), and memory formation in older bees is better than in younger bees (Behrends & Scheiner, 2009; Laloi et al., 2001). Some researchers synchronize worker bee age by enclosing a queen on a comb, subsequently letting bees hatch in the lab and marking them prior to releasing them in their hive until they have the desired age for the experiment (e.g., Groh et al., 2012). While this method allows for standardization of age, other factors that could influence learning capability still vary within the hive. For example, temperature may vary from the edge to the center of the brood nest; 33–36 °C (Kleinhenz et al., 2003). It has been shown that during pupal development temperature affects learning (Jones et al., 2005) and development of the olfactory input region in the brain (Groh, Tautz & Rossler, 2004). Artificial larvae rearing in combination with keeping the emerging bees under laboratory conditions can bypass unwanted environmental effects during formative phases on behavior and brain development. We would also like to point out that testing the effect of different larval food quantities within the hive would heavily be constrained by nurse bee interactions (as in Mattila & Smith, 2008). Indeed, such experiments on individual larvae are only realizable under highly controlled laboratory rearing conditions as described by Aupinel et al. (2007) and Hendriksma, Härtel & Steffan-Dewenter (2011a).

We are the first to our knowledge to investigate learning and memory in combination with how brain structures from artificial reared bees compare with in-hive reared bees. With this set of methods, we were able to evaluate the effect of different quantities of food on brain development. Additionally we evaluated how artificially-reared and artificially-kept bees perform in a differential olfactory conditioning experiment, compared to worker bees from a hive setting (during larval and adult life stages) and found that they are comparable in their performance. Such an experimental set up is not only highly relevant for fundamental research but also for applied research fields such as testing the effects of neuroactive pesticides on bee brain development, learning and memory consolidation. In recent years, the evaluation of sublethal effects on learning and memory (e.g., Ciarlo et al., 2012; Frost, Shutler & Hillier, 2013; Tan et al., 2013; Williamson, Baker & Wright, 2013; Yang et al., 2012) as well as the use of artificial rearing of honey bees (e.g., Charpentier et al., 2014; Hendriksma et al., 2012; Hendriksma, Härtel & Steffan-Dewenter, 2011b; Tavares et al., 2015) are becoming increasingly important tools in the fields of (eco)toxicology and environmental risk assessment. For example, a recent study evaluating sublethal effects of the neonicotinoid thiamethoxam on honey bee larvae reared with the Aupinel protocol could demonstrate changes in the optic lobes but not in other brain structures, like the mushroom bodies (Tavares et al., 2015). However, how artificial rearing itself influences honey bee brain development and learning physiology has so far not been exhaustively studied. In order to utilize in-lab rearing for environmental risk assessment studies, it is important to characterize what impact the rearing method itself has on the analyzed parameters. We have shown that the mushroom bodies, and more specifically the lateral calyces, are smaller at emergence in artificially-reared bees compared to the in hive control. This may impair higher order cognitive functions, such as non-elemental learning tasks (Deisig et al., 2001), which do not become evident when bees are faced with simple associated learning tasks. Whether artificial rearing therefore induces sublethal effects on behavior and cognitive performance thus needs to be further investigated for example by testing bees in complex non-associating tasks like rule learning or positive and negative patterning (as done in Deisig et al., 2001; Sommerlandt, Roessler & Spaethe, 2014).

##  Supplemental Information

10.7717/peerj.3858/supp-1 Supplemental Information 1 Learning curves and retention tests of honeybees that were conditioned in an additional experiment to show that bees could associate either odor equally well with a reward In 12 trials bees were presented with either CS+ or CS − (haphazard order) and PER was recorded. After 1 h and 48 h memory of the conditioned respons was tested (retention). Asterisks show the level of statistical difference between CS+ and CS−, with ***: *p* < 0.001. Regardless which odour was used as CS+ or and as CS−, most bees were able to distinguish odours in the aquisition fase, and during the retention fase. A) Here 1-nonanol was used as CS+ (grey) and 1-hexanol as CS− (black). (B) Here results of the reciprocal conditioning are shown: for CS+ (grey) 1-hexanol was used, and for CS− (black) 1-nonanol was used.

10.7717/peerj.3858/supp-2 Supplemental Information 2 Acquisition data, or PER data during acquisition phase (the learning phase) of the olfactory conditioning experiment

10.7717/peerj.3858/supp-3 Supplemental Information 3 Bee brain data used for the comparison of different brain neuropils

10.7717/peerj.3858/supp-4 Supplemental Information 4 Data on intertegula span and head width

10.7717/peerj.3858/supp-5 Supplemental Information 5 Retention data, or PER during the 1 h and 48 h retention tests (part of the olfactory conditioning experiment)

## Additional Information and Declarations

### Data Availability

The following information was supplied regarding data availability: The raw data has been provided in the Supplemental Files.
