# Supplementary figures and images for: Learning performance and brain structure of artificially-reared honey bees fed with different quantities of food
Source: PeerJ. 2017 Oct 23;5:e3858. doi: 10.7717/peerj.3858 (PMC5657415; doi:10.7717/peerj.3858)

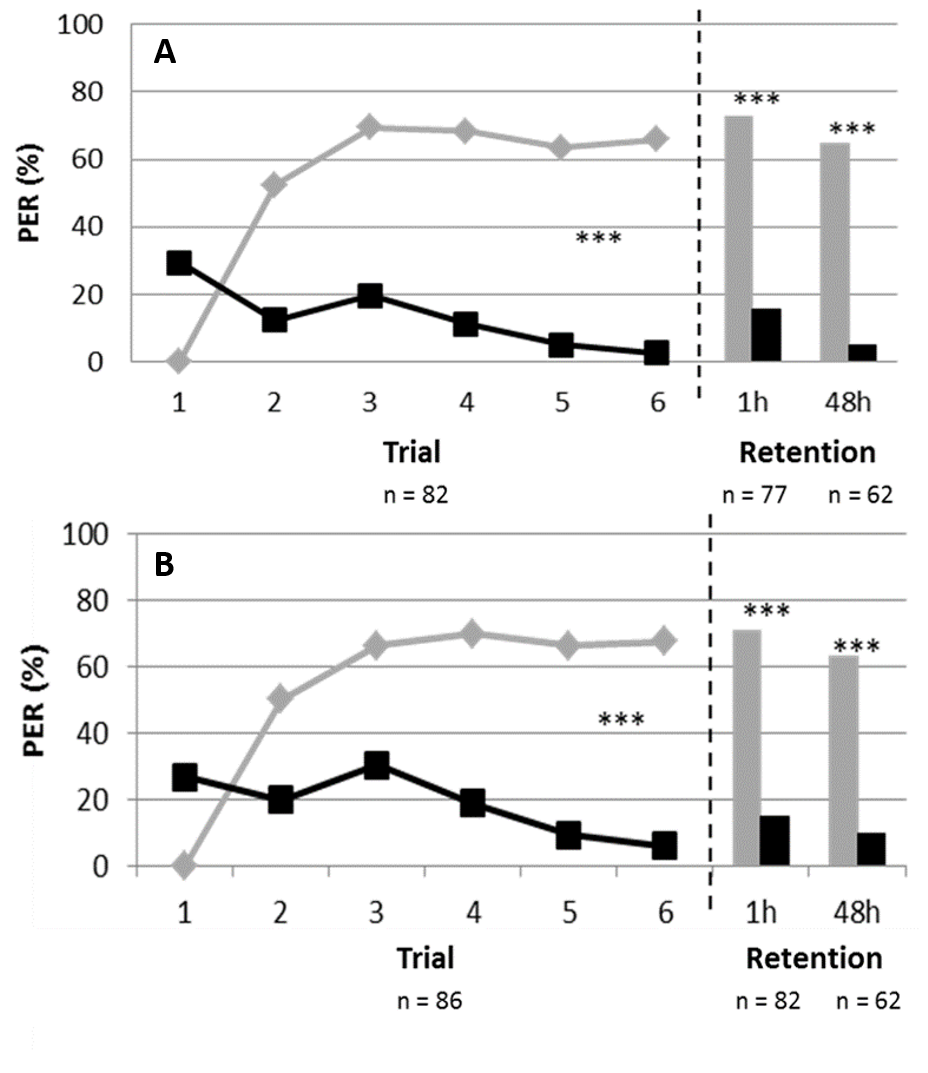

Supplement: Supplemental Information 1 — In 12 trials bees were presented with either CS+ or CS − (haphazard order) and PER was recorded. After 1 h and 48 h memory of the conditioned respons was tested (retention). Asterisks show the level of statistical difference between CS+ and CS−, with ***: p < 0.001. Regardless which odour was used as CS+ or and as CS−, most bees were able to distinguish odours in the aquisition fase, and during the retention fase. A) Here 1-nonanol was used as CS+ (grey) and 1-hexanol as CS− (black). (B) Here results of the reciprocal conditioning are shown: for CS+ (grey) 1-hexanol was used, and for CS− (black) 1-nonanol was used. [file peerj-05-3858-s001.png]
